# Jan Piltz (1870–1930)

**DOI:** 10.1007/s00415-015-7695-6

**Published:** 2015-03-13

**Authors:** Anita Magowska

**Affiliations:** Poznań University of Medical Sciences, Poznań, Poland

**Fig. 1:**
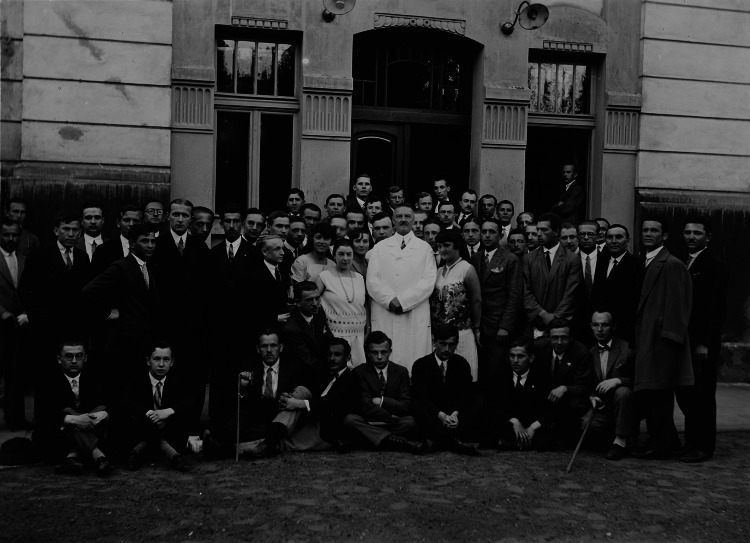
Professor Jan Piltz (in the *middle*, in a *white* coat) and his assistants in front of the seat of the Clinics of Neurology and Psychiatry, Cracow, 1928

Born in Russian Poland (at that time Poland was partitioned by Russia, Austro-Hungary and Prussia), Jan Piltz (1870–1930) graduated in medicine from the University of Zurich in 1895. His internship was served with Eugen Bleuler at the famous University Psychiatric Clinic in Burghőlzli close to Zürich (‘Burghőlzli’), then headed by Auguste Forel. However, Piltz felt himself as being Polish and intended to practice medicine in his homeland, hence, he had his Swiss diploma at the University of Kazan in Russia approved, and in 1897 took a doctorate under Bekhterev in St. Petersburg. Nonetheless, when his friend Bleuler became director of the Burghőlzli and invited him to reorganize its internal structure, Piltz returned to Switzerland. He turned out to be such a flexible manager that after 2 years the cantonal government of Vaud appointed him as deputy director at the psychiatric clinic in Lausanne, at the time headed by Albert Mahaim [1] (Fig. 1).

The Zurich period was the most important time in Piltz’s life. Then his research profile was enriched by Bleuler’s clinical experience and concern to explain mental illnesses in terms of affects and psychogenesis. Bleuler encouraged him to investigate the diagnostic value of pupillary responses in mental illnesses; the concept was highly appreciated by Charcot and Forel but was then without any practical meaning. Piltz observed the dilation of the pupil in response to light and visual stimuli in hospitalized patients and compared the results to the changes of the pupil which appeared while faradizing the cerebral cortex of animals [2]. In 1899, he described a peculiar disturbance in the innervation of the musculus *sphincter iridis* simultaneously with Alexander Westphal [3]. The phenomenon manifests in the constriction of the pupil in the case of voluntary contraction of the *orbicularis oculi* muscle and is known as the Westphal–Piltz neurotonic pupillary reaction. However, as Piltz emphasized in his article, it had already been observed by Wundt, Giffard and Galassi [4]. He also found that when attention was turned to a dark object within the field of vision whilst illumination remained unchanged an expansion of the pupils takes place. This phenomenon, together with the previously described observation of Otto Haab, is known as the Haab–Piltz attention reflex. The influence of cognitive load on the pupil manifested even in blind persons, which Piltz observed in 1899 in an asylum for the blind and deaf in Zurich [5].

In 1900, Piltz finally left Zurich to practice under Jules Déjerine and Józef Babiński at the Hopital Salpetrière in Paris and, enabled by Bleuler, to visit the most prestigious psychiatric clinics in Germany, the Netherlands, France, and Russia. Two years later, he was appointed organizer and physician-in-chief at the first neurological department of the city hospital in Warsaw. Apart from hospital wards, Piltz organized an operating room for physiological experiments on animals [1]. There he engaged in research on the topography of cortical pupillary motor centres. He determined that irritation with very weak currents brought forth quite isolated pupillary movements which are not accompanied by movements of the eyeball and eyelids. He took into consideration only isolated pupillary movements, which earlier investigators such as Bekhterev, Brown-Séquard, Ferrier, and Horsley had not considered [6].

Piltz arranged suitable physiological experiments on dogs to analyse the frequency of pupillary symptoms in nervous diseases. With an inductive current he sought out the cortical zones which determine eye movements in dogs, removed them and then studied the secondary degeneration by the Marchi method. In this way, he could determine nerve tracts inside the brain. Furthermore, after exposing the long and short ciliary nerves he irritated them with an electrical current. By isolated irritation in a single ciliary nerve or by the simultaneous irritation of several of these nerves, he succeeded in artificially producing all possible combinations of irregularities in the contour of the pupil as they appear in the course of nervous disease [7]. To represent graphically the physiological phenomena of the pupils and their pathological disturbances, in 1904 Piltz designed a pupillograph which consisted of a movie camera, a long lens, and a lamp powered by storage batteries [8].

In 1905, Piltz was appointed assistant professor of the Jagiellonian University with the task to organize the first Polish neurological-psychiatric teaching hospital. He patterned the hospital on the Swiss ones. Before the buildings were completed, which occurred just before the outbreak of the Great War, he focused on the proper training of medical staff, therefore he sent his future assistants as interns to the Burghőlzli, and psychiatric hospitals in Lausanne, Rheinau, Zurich, and Munich. After the outbreak of the Great War, the hospital was turned into a military ward of the fortress hospital in Cracow where about 11,000 servicemen, including 3000 with war neurosis, were treated. With the coming of peace, the ward was reorganized into a teaching hospital again, still headed by Piltz, then a full professor, and the first generation of Polish neurologists and psychiatrists were trained there [9]. He died prematurely in 1930 in Cracow due to postoperative complications [1].
